# Development of a Prediction Model to Identify the Risk of *Clostridioides difficile* Infection in Hospitalized Patients Receiving at Least One Dose of Antibiotics

**DOI:** 10.3390/pharmacy12010037

**Published:** 2024-02-19

**Authors:** Abdulrahman Alamri, AlHanoof Bin Abbas, Ekram Al Hassan, Yasser Almogbel

**Affiliations:** 1 Pharmaceutical Care Services, Ministry of the National Guard Health Affairs, Riyadh 11426, Saudi Arabia; 2 King Abdullah International Medical Research Center, Riyadh 11481, Saudi Arabia; 3 Department of Pharmacy Practice, College of Pharmacy, King Saud bin Abdulaziz University for Health Sciences, Riyadh 11481, Saudi Arabia; 4 Department of Pharmacy Practice, College of Pharmacy, Qassim University, Buraidah 51452, Saudi Arabia; alhanoofbinabbas@hotmail.com (A.B.A.); y.almogbel@qu.edu.sa (Y.A.); 5 Department of Pathology and Laboratory Medicine, Ministry of the National Guard Health Affairs, Riyadh 11426, Saudi Arabia; hassaini@ngha.med.sa

**Keywords:** model, clostridium difficile infection, clostridioides difficile infection, risk prediction, clostridium difficile infection risk factors, chronic kidney disease

## Abstract

Objective: This study’s objective was to develop a risk-prediction model to identify hospitalized patients at risk of Clostridioides difficile infection (CDI) who had received at least one dose of systemic antibiotics in a large tertiary hospital. Patients and methods: This was a retrospective case–control study that included patients hospitalized for more than 2 days who received antibiotic therapy during hospitalization. The study included two groups: patients diagnosed with hospital CDI and controls without hospital CDI. Cases were matched 1:3 with assigned controls by age and sex. Descriptive statistics were used to identify the study population by comparing cases with controls. Continuous variables were stated as the means and standard deviations. A multivariate analysis was built to identify the significantly associated covariates between cases and controls for CDI. Results: A total of 364 patients were included and distributed between the two groups. The control group included 273 patients, and the case group included 91 patients. The risk factors for CDI were investigated, with only significant risks identified and included in the risk assessment model: age older than 70 years (*p* = 0.034), chronic kidney disease (*p* = 0.043), solid organ transplantation (*p* = 0.021), and lymphoma or leukemia (*p* = 0.019). A risk score of ≥2 showed the best sensitivity, specificity, and accuracy of 78.02%, 45.42%, and 78.02, respectively, with an area under the curve of 0.6172. Conclusion: We identified four associated risk factors in the risk-prediction model. The tool showed good discrimination that might help predict, identify, and evaluate hospitalized patients at risk of developing CDI.

## 1. Introduction

Clostridium difficile infection (CDI) or *Clostridioides difficile* infection carries a high risk, especially in the elderly and in patients with gut microbiota dysbiosis resulting from antibiotics exposure [1,2]. Within healthcare facilities, the transmission usually occurs through the hands of staff who are contaminated with *Clostridioides difficile* spores or from environmental contamination. This could include occupying a room where a prior occupant had CDI [3,4,5,6,7]. Due to limited treatment options and the absence of primary prevention for CDI, it is essential to understand the multiple factors that influence the risk of developing it. Commonly reported risk factors include, but are not limited to, advanced age; coexisting conditions like chronic kidney disease (CKD), diabetes mellitus, inflammatory bowel disease, and cancer; use of medication such as antibiotics, histamine-2 receptor antagonists, and proton pump inhibitors (PPIs); and exposure to healthcare settings (recent hospitalization). Other risk factors reported in the literature include obesity, surgery, use of non-steroidal anti-inflammatory drugs, enteral feeding, cancer chemotherapy, hematopoietic stem cell transplantation, and liver cirrhosis [8,9]. Because of the increasing frequency of CDI and its corresponding mortality, strategies to prevent or stop CDI have gained attention. Several strategies are possible, such as replacing normal flora with probiotics, enhancing infection control policies, and minimizing the use of PPIs [10]. It is necessary to assess additional factors, such as the use of broad-spectrum antibiotics, in order to identify patients who are most likely to develop CDI. It would be helpful to have a clinical risk model that predicts which patients are most likely to suffer with the illness based on established CDI risk factors. There are few risk-prediction tools in the literature to identify these patients who are admitted to hospital. Applying most of these tools has been challenging because of difficulties in measuring certain variables included in these models [2,3,8,9,10,11,12,13]. There are a few variations between these models that have been noted. There are some restrictions on the use of each model at the time of hospital admission. Certain models look into multiple variables, some of which may not be known until hospitalization or are not regularly collected. A study by Oh et al., conducted in 2018, used the data from two large academic centers to develop two risk-stratification models specific to institutions for the prediction of daily risk of CDI. According to the study’s results, the models exhibited a high capacity to identify CDI-risk patients in each specific institution. However, this approach may allow early detection of individuals who are highly susceptible to CDI, and only the cases that are identified during the current admission are included [13]. The aim of the study was to identify previously unidentified variables predicting incidence of *Clostridioides difficile* infection in adult patients and to propose a predictive model based on these results. 

This study’s objective was to develop a risk-prediction model to identify hospitalized patients at risk of CDI who have obtained a minimum of one dose of antibiotics systematically.

## 2. Materials and Methods

### 2.1. Study Design and Setting

A retrospective case–control study was carried out between 1 January 2016 and 31 July 2018. Based on age (±5 years) and gender, each patient in the case group was matched with three patients in the control group.

King Abdulaziz Medical City, Ministry of National Guard Health Affairs (NGHA), was the site of the research. Riyadh, Saudi Arabia, is a tertiary hospital with 1973 operational beds. The medical city provides all levels of care, including public health, primary healthcare, and all specialized care and tertiary services. It contains several centers, such as an organ transplant center, cardiovascular center, and oncology center. In addition, it also has King Abdullah Specialized Children’s Hospital, which is a specialized referral hospital for children. There are currently 5282 administrative and support professionals and 8584 allied health and medical support professionals on staff, in addition to the 2451 physicians, dentists, and residents [14].

### 2.2. Inclusion Criteria

Cases: We included every patient admitted, who is at least 18 years old, for more than 2 days who, while in hospital, had at least one systemic dose of antibiotic therapy and had either a positive stool sample culture, a positive polymerase chain reaction result, or a positive *Clostridioides difficile* toxin assay result. For any patient hospitalized within 28 days following an earlier admission, the prior admission data were included [15]. Any previous exposure to antibiotics was described as receiving one dose of antibiotics any day for the four weeks prior to the diagnosis of CDI [16].

Controls: All patients ≥18 years who were admitted for more than 2 days to the hospital during the study period, who received systemic antibiotic therapy during hospitalization and tested negative for CDI, were matched to cases with their age and sex.

### 2.3. Exclusion Criteria

Individuals who were diagnosed with CDI within 48 h of hospital admission or who were admitted for less than 48 h were not included in the Electronic Medical Record (EMR) to minimize the cases of community-acquired CDI [15,16,17]. We also excluded subsequent readmissions of CDI-positive patients, patients with CDI that existed prior to the start of hospital antibiotic treatment, pregnant women aged younger than 18 years, patients with incomplete data in the hospital systems, or if no antibiotic was administered.

### 2.4. Data Collection Procedures, Data Sources, and Measurements

The information technology department provided all the adult patients’ lists during the study period. The clinical microbiology laboratory provided the *Clostridioides difficile* toxin testing date and results for each patient who was tested for CDI. The clinical information was gathered and examined from the EMR, BESTCare. Each patient’s data were entered into a separate approved data collection form. Each patient’s EMR was consulted for the following: demographic data, comorbidities, and previous laboratory data within 3 months. We considered renal disease as creatinine clearance (CrCl) ≤ 30 mL/min/1.73 m^2^. All data from the data collection sheet were protected in a secured drawer, to which only the researchers had access. Furthermore, we gave every medical record a special serial number to ensure strict adherence to confidentiality and comprehensive privacy.

### 2.5. Data Management and Analysis

Data management and analysis were performed using Excel 2016 to collect data, and STATA version 16 was employed for carrying out each statistical analysis. The study population was described using descriptive statistics. The means and standard deviations were used to express continuous variables. Student’s *t*-tests were then performed to compare means between the study groups. The chi-square test was used to analyze the categorical variables, which were measured in frequencies. To find the covariates that were significantly associated with CDI between the cases and controls, a multivariate analysis was performed. To determine the variables connected to CDI, logistic regression and conditional logistic regression have been used. Then, logistic regression was employed in the final analysis which included any variables at hospital admission with a *p*-value ≤ 0.2 to identify factors associated with CDI [18]. The final regression model was converted into a point-based instrument, and the variables found to be connected with CDI were given weighted scores. By dividing each regression coefficient by half of the smallest coefficient and rounding it to the nearest integer, the scores allocated to each variable were calculated [19]. At different tool point cutoffs, the tool’s positive predictive value (PPV), negative predictive value (NPV), sensitivity, specificity, and accuracy were measured and assessed. In order to evaluate the discrimination powers of the risk model, a receiver operating characteristic area under the curve (ROC-AUC) was also built.

### 2.6. Ethics Approval

The Institutional Review Board (IRB) of the King Abdullah International Medical Research Center in Riyadh, Saudi Arabia, approved this study after reviewing the proposal submission. (IRB registration number: H-01-R-005). The need for a study using anonymous data and the need for consent for a retrospective study have been waived by the IRB; however, the approval of the research protocol by the IRB including the permission from the authority of the hospital to review the patients’ records has been obtained. If any organs were donated, they were done so willingly and in compliance with the Istanbul Declaration, having provided written informed consent. Everyone’s informed consent was acquired.

## 3. Results

**Figure 1:**
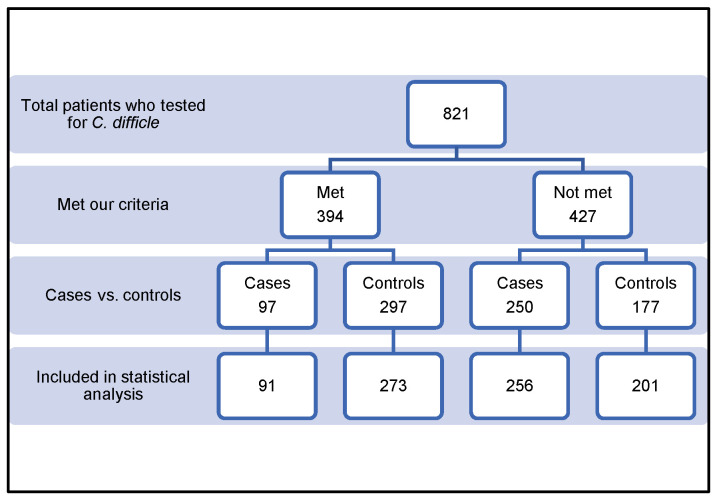
Inclusion flowcharts of cases and controls.

We screened and reviewed a total of 821 patients. Of these, 457 patients were excluded for various reasons, as summarized in Table 1. The remaining 364 patients were included in the study, as shown in Figure 1. Patients were allocated into two groups: the case group (n = 91, 25%) and the control group (n = 273, 75%). All demographic and inferential data were collected and are outlined in Table 2. The mean ± SD age of the study population was 62.76 ± 18.5 years. The mean ± SD age for the group under control was greater than that for the case group, 61 ± 18.5 and 67.5 ± 19.6, respectively. Over fifty-two percent of the patients in the case group had an age of ≥70 years, as opposed to less than 33% in the control group (*p* = 0.024). A statistically significant correlation was found between advanced age (≥70 years) and CDI development. The study population was 48.4% male and 51.6% female. The development of CDI was not statistically significantly correlated with sex. The mean body mass indexes ± SD in the case group and control group were comparable (26.5 ± 7.08 and 25.01 ± 7.4, respectively) (*p* = 0.087). The total duration of stay did not significantly differ between the two groups. The association of various comorbidities with CDI is summarized in Table 3. The associations between type of surgery and CDI development in the study groups are shown in Table 4. The two groups did not significantly differ from one another regarding the use of PPI, ranitidine, or statin (Table 5). In the final analysis, any variable included in the final multivariate logistic regression analysis had a *p*-value of less than or equal to 0.2 (Table 6). Age older than 70 years (*p* = 0.034), CKD (CrCl < 30 mL/min) (*p* = 0.043), solid organ transplantation (SOT; *p* = 0.021), and lymphoma or leukemia (*p* = 0.019) were discovered to be significantly correlated with CDI in hospitalized patients. The calculated risk score was two for each (Table 7).

**Figure 2:**
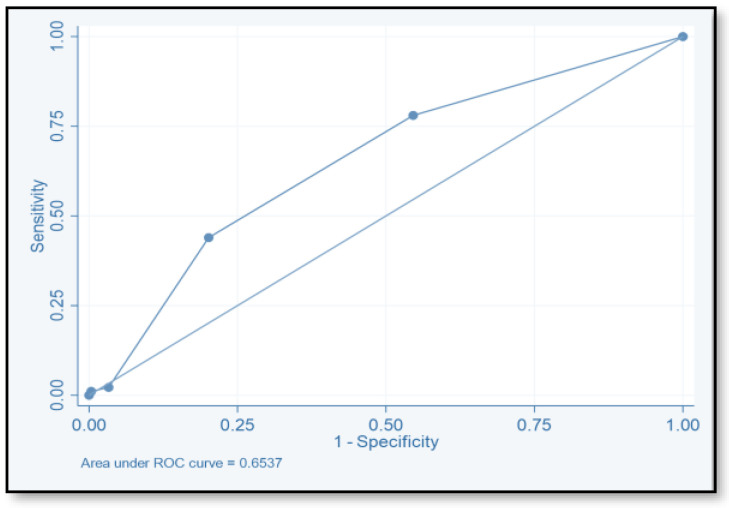
Receiver operating characteristic for the total score.

**Figure 3:**
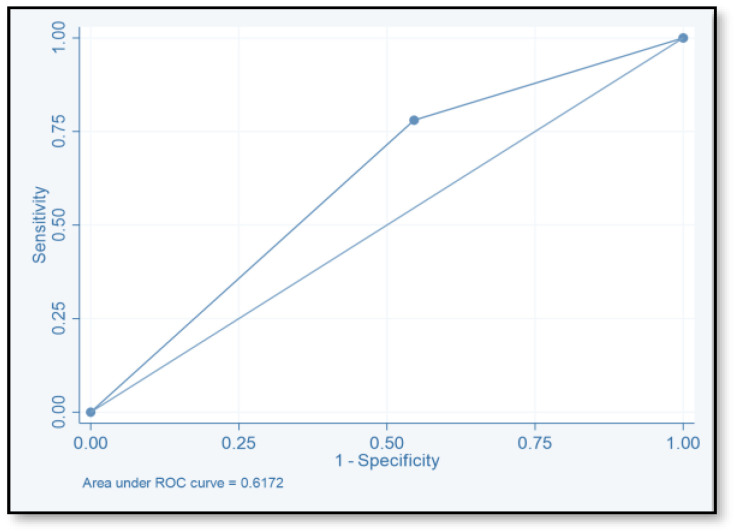
Receiver operating characteristic curve of score ≥2.

**Figure 4:**
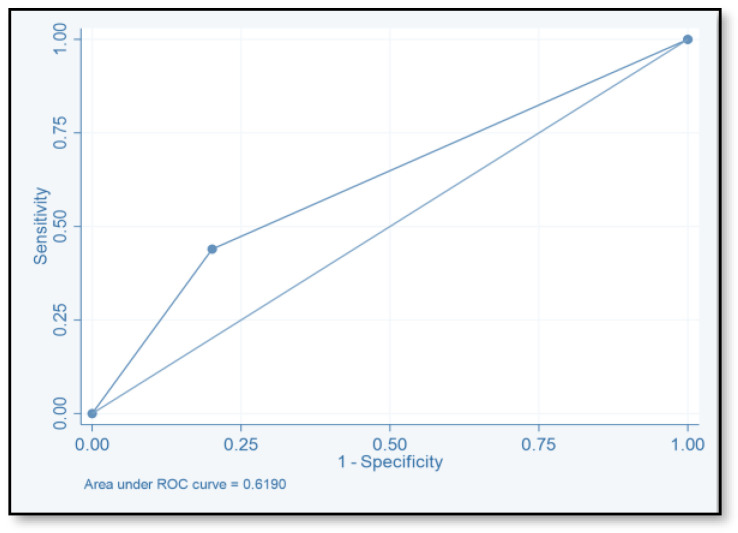
Receiver operating characteristic curve of score ≥4.

**Figure 5:**
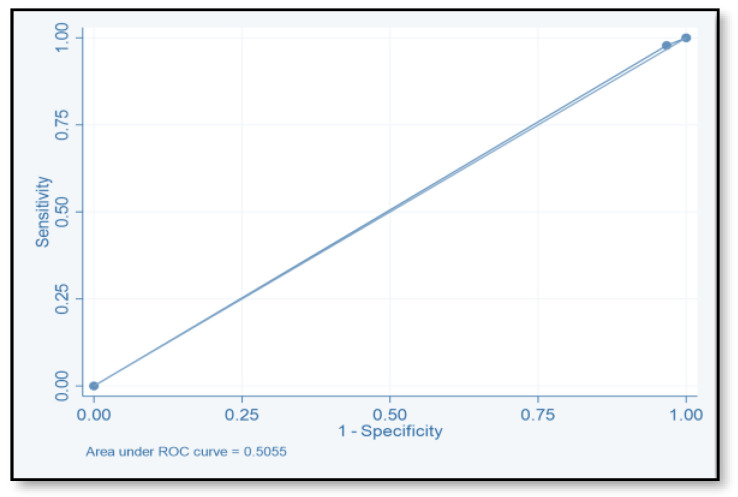
Receiver operating characteristic curve of score ≥6.

**Figure 6:**
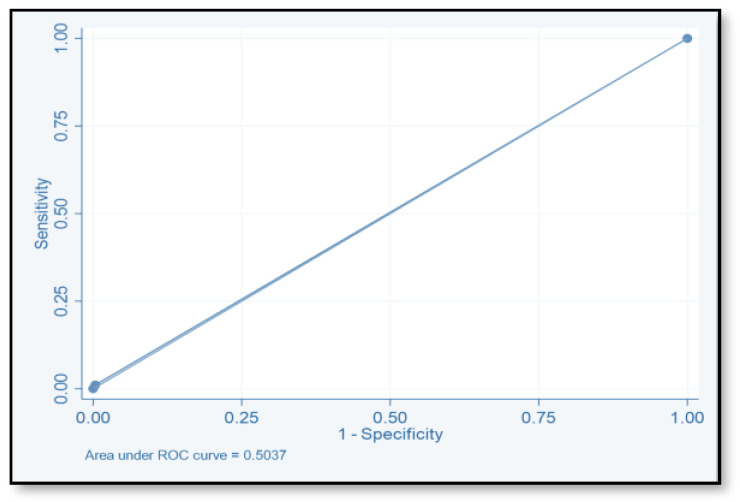
Receiver operating characteristic curve of score ≥8.

The discriminative ability of the risk assessment model has been estimated using an ROC-AUC of 0.653 (Figure 2). The accuracy, PPV, NPV, sensitivity, and specificity of scores were investigated (Table 8). The highest accuracy was observed with a score of ≥2 and with an ROC-AUC of 0.617 (Figure 3). At a score of ≥4, the accuracy was 43.96, with 43.96% sensitivity and 79.85% specificity, respectively, with an ROC-AUC of 0.619 (Figure 4). At a score of ≥6, the accuracy was only 2.2, with 96.7% specificity and 2.2% sensitivity, respectively, and an ROC-AUC of 0.5055 (Figure 5). At a score of ≥8, the accuracy was only 1.1, with a sensitivity and specificity of 1.1% and 99.6%, respectively, with an ROC-AUC of 0.5037 (Figure 6).

## 4. Discussion

Earlier studies have critically evaluated and found a number of important patient risk factors for CDI. The incidence of CDI has been found to be strongly correlated with a number of independent risk factors, although some studies have reported contradictory results regarding those risk factors. Our study’s findings revealed associations between CDI and patients with advanced age, history of kidney dysfunction, and who are immunocompromised. There were no discernible variations between the study groups regarding the presence of other comorbidities and patient characteristics.

### 4.1. Risk factors

#### 4.1.1. Age

Our results revealed findings similar to the previously published information on the impact of age on CDI development. According to our data, the patients with CDI had an average age of 67.5 ± 19.6 years (±SD), and the risk of CDI increased by approximately 2-fold in patients aged ≥ 70 years. These findings match the outcomes of a retrospective case–control, multicenter study from July 2015 to July 2017, conducted by Tilton and Johnson. Every adult patient who had taken systemic antibiotics at least once was included in the study. The findings indicated that a higher risk of hospital-onset CDI had been linked to advanced age (≥70 years) (adjusted odds ratio [OR] 1.89; 95% confidence interval [CI) 1.05–3.43; *p* = 0.0326) [19]. Another investigation, performed between 2008 and 2012, was conducted by Patel et al. of a veteran population aged ≥18 years with documented CDI. Their results revealed that the age of ≥70 years as associated with severe CDI-related risk. Forty three percent of patients with severe CDI overall and 23% of patients with non-severe CDI were 70 years of age or older (*p* = 0.004). A logistic regression analysis was used to confirm this outcome even further. PPI use, three or more antibiotic prescriptions, and patient age of ≥ 70 years were included in the logistic regression model. This model took into account antibiotics and PPI use and showed that being over 70 is one of the independent, statistically significant risk factors for severe CDI, (OR 2.39; 95% CI 1.28–4.50; *p* = 0.006) [20]. In addition, a 1-year retrospective chart review was conducted by Henrich et al. between June 2005 and May 2006, which included all inpatients aged ≥ 18 years who had a positive fecal *Clostridioides difficile* toxin result, to evaluate patient and clinical characteristics linked to severe diarrhea caused by clinical characteristics linked to severe diarrhea caused by Clostridium difficile. An age of 70 years was found to be a risk factor for severe CDI (OR 3.35; 95% CI 1.42–7.38; *p* = 0.001) [21].

#### 4.1.2. Kidney Dysfunction

The results of this analysis indicated that patients with CKD (CrCl < 30 mL/min) were at risk for CDI development. Hospitalized patients with CKD had a greater than two-fold increased risk of developing CDI compared to those without CKD, as indicated by Table 7. Although the mechanism of CKD increasing the risk of CDI is unclear, a few studies have reported that CKD causes chronic systemic inflammation that leads to the acquired immunodeficiency that makes patients with CKD more vulnerable to CDI [22,23]. Other authors have attributed this increased risk to the microorganisms’ overgrowth or gastric suppression resulting from reduced intestinal motility, which is typically observed in patients with CKD [24,25]. Evaluation of CKD as a risk factor for CDI has been completed in the previous literature and linked to increased mortality in patients with CDI. Keddis and colleagues carried out research to look into the association between hospital outcomes and CDI in CKD patients using a cohort approach. The data were obtained between 2005 and 2009 via the National Hospital Discharge Survey. More than 8 million patients with CKD were identified. Compared to inpatients without CKD, the CDI rate was 1.49% (*p* = 0.001). Moreover, the findings showed that compared to people who did not receive regular dialysis, dialysis patients had a 1.33-fold increased risk of developing CDI. This number increased to more than twice that of patients with no CKD. They also found that infection with CD in a patient with a history of CKD was associated with a longer hospital stay, a high colectomy rate, and a rise in death rate within hospitals (OR 1.55; 95% CI 1.52–1.59; all *p* = 0.001) [26]. A study was conducted among Korean patients in a retrospective case–control design matched using age and sex, comprising 171 individuals with a verified diagnosis of CDI and 342 patients without CDI. They reported that the patients with advanced CKD (with a reduced glomerular filtration rate < 60 mL/min), compared to those without CKD, those with end-stage kidney disease, among others, have a higher chance of developing CDI and are more likely to experience CDI than people without CKD (OR 2.10; *p* = 0.003). Furthermore, CKD can increase mortality and poor response to initial therapy in hospitalized patients [27].

#### 4.1.3. Lymphoma/Leukemia

Our results agreed with the previously published data, which demonstrated that patients had a higher risk of developing CDI if they had a history or current diagnosis of lymphoma or leukemia. There was a significant increase in CDI risk that was approximately four times higher among patients with lymphoma or leukemia. Several studies have addressed these findings in various patient populations.

A retrospective cohort study conducted among Australian patients between July 2011 and June 2012 included every patient brought into hospitals with a hematological cancer. According to the study, patients with bacterial pneumonia or another bacterial infection, as well as those with acute lymphocytic leukemia and neutropenia, were at risk of CDI. They also reported that a higher risk of death within 60 and 90 days following CDI was linked to CDI in these populations [28].

Anjali Bal et al. conducted a retrospective review to ascertain the CDI rate within ninety days of beginning chemotherapy in those suffering from acute myeloid leukemia (AML) between 2011 and 2016, and they assess the features, comorbidities, risk factors, and CDI-related disease-specific indices of the patients. They found that 31% of the patients with AML had CDI, 15.9% had at least one recurring episode of CDI, and 4.5% had multiple instances. More than 40% of the patients underwent a computed tomography scan that revealed that 6.8% of the patients had typhilitis and (4.5%) had a toxic megacolon and underwent colectomy [29].

A prospective Cologne cohort study has been conducted to evaluate the risk factors associated with CDI in patients receiving treatment for acute myeloid leukemia (AML) and receiving allogeneic stem cell transplantation (aSCT), as well as the epidemiology and management of CDI in these patients. The investigators reported that for patients with AML and recipients of an aSCT, the CDI incidence rates per 10,000 patient days were 17.9% and 27.4%, respectively. In contrast, the incidence rates were 6.1% for patients with AML and 12.6% for aSCT recipients for CDI per hospitalization. Carbapenems were recognized as one of the main risks for CDI. In addition, it was thought that having a Clostridium difficile infection increased the chance of developing acute gastrointestinal graft versus host disease [30]. The study’s results revealed that patients with a severe immunosuppressive state and high antibiotic exposure were high-risk populations for CDI, with rates per hospitalization of 4.8–9.3% and 12.5–30%, respectively [31,32,33,34].

#### 4.1.4. Solid Organ Transplant

The study results revealed that patients who underwent SOT had a 3-fold higher CDI risk than those who did not undergo SOT. In our study, we aimed to investigate the occurrence of *Clostridioides difficile* infection (CDI) in patients who underwent organ transplantation. The time interval between transplantation and CDI development, as well as the association between specific types of transplanted organs and CDI, have not been thoroughly explored. However, according to the earlier research, the majority of CDI cases happen in the first month following transplantation [35,36]. Paudel et al. recently conducted a meta-analysis, looking at 30 studies that evaluated CDI among SOTs between 1991 and 2014. This meta-analysis reviewed over 21,000 patients. All the patients underwent one or more organ transplants, including lung, kidney, heart, liver, pancreas, or intestine. The authors reported that in SOT patients, the overall prevalence of CDI was 7.4% (95% CI 5.6–9.5%). The rate of infection varied according to the kind of transplanted organ. The highest CDI prevalence was among patients who had received more than one organ transplant, 12.7% (95% CI 6.4–20.9%). The remaining organs were: kidney 4.7% (95% CI 2.6–7.3%), pancreas 3.2% (95% CI 0.5–7.9%), lung 10.8% (95% CI 5.5–17.7%), liver 9.1% (95% CI 5.8–13.2%), intestine 8% (95% CI 2.6–15.9%), heart 5.2% (95% CI 1.8–10.2%), and intestine 8% (95% CI 2.6–15.9%) [37]. Another single descriptive study conducted by Tsapepas et al. between September 2009 and December 2012 discovered a 4% overall CDI incidence among SOTs, with lung transplant recipients experiencing the highest rate. The incidence of CDI in recipients of liver, kidney, lung, and heart transplants was 2.7%, 3.2%, 1.9%, and 7%, respectively (*p* = 0.03 between organ types) [38].

### 4.2. Risk Prediction Model

The final regression model for the factors associated with CDI among hospitalized patients was changed into a point-based instrument where the variables linked to hospital-onset CDI were given weighted scores. By dividing each regression coefficient by half of the smallest coefficient and rounding to the nearest whole number, the scores allocated to each variable were a calculated integer [19,39]. Several cutoffs were considered at scores of 2, 4, 6, 8, 10, and 12. The maximum score was 12, with ages older than 70 years and the presence of renal disease scored 2 for each risk factor, whereas SOT and lymphoma or leukemia scored 4 for each risk factor. We looked into and obtained the sensitivity, specificity, PPV, NPV, and accuracy.

To evaluate the model’s discriminative ability to correctly classify patients at risk of CDI, we determined the ROC-AUC by displaying the true-positive rate (sensitivity) versus the false-positive rate (specificity = 1). We also determined the ROC-AUC for each cutoff point value of the risk assessment model. The ability of this model to predict the CDI risk has been estimated (ROC-AUC values of 0.653). The best ROC-AUCs of 0.6172 and 0.619 were obtained for scores ≥ 2 and ≥ 4, respectively. The ROC-AUCs of other published models were 0.70–0.88 [16,17,18,40]. We believe that this model could be used easily by healthcare practitioners even at the time of admission. Tanner and his colleagues used the Waterlow score to predict which patients, upon hospital admission, would develop an illness associated with Clostridium difficile. This pressure ulcer risk scale is used to evaluate a patient’s potential for developing one. Their model’s usability for hospitals that are already calculating the Waterlow score is a major benefit. Dubberke and his colleagues intended to create and verify the risk-prediction model for CDI. The main limitation of their model is that the risk-prediction model was created using complex statistics, and some of the model’s variables were not easily accessible. (e.g., modified APS and CDI pressure) [17]. Other risk-prediction tools in the literature also have limits when used as a preliminary screening instrument on hospital admissions and in obtaining some of the variables.

We believe that this model could be employed to recognize those patients with a high chance of suffering CDI in a hospital setting; to accomplish this, we should select a threshold value that is highly sensitive (ie score ≥ 2, sensitivity = 78.02). Another application for this score could be identifying patients needing CDI prophylaxis or prevention measures for recurrence of the infection, particularly in patients undergoing CDI treatment and those who have a high chance of recurrence. Hence, we should use the cutoff score that sacrifices reduced sensitivity in favor of greater specificity and PPV (i.e., score) ≥ 8, SP = 99.6, and PPV = 50. The Infection Disease Society of America (IDSA) recommends against the use of routine pharmacological prophylaxis for CDI. However, Bezlotoxumab, the first FDA-approved monoclonal antibodies, is suggested as a new agent to be used for the prevention of recurrent CDI [41]. According to IDSA guidelines, bezlotoxumab should be used in addition to standard antibiotics against CDI in individuals who have advanced age, immunocompromised status, or a history of severe CDI42 as risk factors for CDI recurrence. Additional precautions should be taken, such as the use of sterile tools and devices, personal protective equipment, and infection-control prevention techniques like proper hand hygiene, proper patients’ room hygiene, and an established antimicrobial stewardship program. Further studies need to be conducted to evaluate the application of this risk assessment model to hospitalized patients

To the extent of our understanding, this, we think, is the first model that has been examined and developed in hospital settings in Saudi Arabia. The best way to apply and validate this model to identify individuals at risk of CDI should be determined as the next course of action.

### Strengths and Limitations of the Study

Infectious disease epidemiology has not yet made extensive use of risk-prediction modeling, particularly for CDI, to develop a tool that could identify people who are susceptible to CDI. The ultimate objectives are to support healthcare providers in their decision making, enable patients to learn about their conditions, and recognize and implement preventive measures. Hospitalization variables, certain comorbid conditions, and the use of broad-spectrum antibiotics are the most common causes of CDI in hospitalized patients [42,43,44]. We have developed a practical CDI risk-prediction model to accurately determine which patients are at CDI risk with specific management strategies, which will result in better management, reduced morbidity and mortality, and a shorter duration of hospital stay. We believe that this is the first risk-prediction-model development conducted in the Middle East that could enhance clinical judgment, the distribution of resources for CDI stewardship programs, and the design of research with a recognized predictive performance and calibration. Every variable contained in this risk-prediction model is easily accessible in the electronic patient file upon admission, and it is quick and easy to calculate the risk for each patient.

There are limitations to this study. Since this is a retrospective study, there was bound to be some missing documentation. In this study, every patient came from the same single facility, although it is one of the larger hospitals in Riyadh, Saudi Arabia. Multicenter studies might be able to report more data and help validate this model. The model had low performance ability. Although the ROC curve has performed better with fewer predictors, adding more predictors showed a lower discrimination level. Using more predictors in the model requires a better sample size. The number of cases was a significant problem. All the patients available in the hospital were utilized. There are different approaches to a study analysis; logistic regression was used in this study because it has been reported in the literature and in many other studies with the same design as ours [15,17,19]. Others, on the other hand, used conditional logistic regression, which may be considered the optimal analysis method. Lastly, After validation, the risk levels found in this population could be utilized to determine prevention strategies that are affordable and tailored to the specific risk levels of each group.

## 5. Conclusions

This study identified a significant correlation between four risk variables that are part of the developed risk-prediction model, including older age, CKD, SOT, and lymphoma or leukemia, which increased the risk for CDI. This tool could help predict and identify those hospitalized patients at risk of developing CDI. It is also helpful for early initiation of preventive prophylaxis measures for CDI including contact isolation and other empirical precautions.

## Figures and Tables

**Table 1:** Reasons for exclusion from the study.

Reason	No.
Age < 18 years	210
Incomplete data	16
No previous admission	130
No antibiotics were administered	57
Hospitalization < 48 h	10
Pregnant woman	2
Recurrent *Clostridioides difficile* infection	2
Not matched	30

**Table 2:** Distribution of demographics and association between variables among cases and controls.

Characteristics			Chi-Square Analysis				Conditional Logistic Regression	
			Controls n (%) 273 (75.0)	Cases n (%) 91 (25.0)	Total n (%)	*p*-Value	OR (95% CI)	*p*-Value
Age groups		<70	166 (60.8)	43 (47.3)	209 (57.3)	0.024	1 (matched)	1.000
		≥70	107 (39.2)	48(52.8)	155 (42.7)			
Age (years) mean ± SD			61 ± 18.5	67.5 ± 19.6				
Age mean cases + control			62.76 ± 18.5 (18.96)					
Sex		Male	132 (48.4)	44 (48.4)	176 (48.4)	1.000	1 (matched)	1.000
		Female	141 (51.6)	47 (51.6)	188 (51.6)			
Mean body mass index (kg/m^2^)			26.50 ± 7.08	25.01 ± 7.4	–	0.087	0.97 (0.93–1.01)	0.070
Creatinine clearance			62.2 ± 43.0	53.6 ± 37.5	–	0.088	0.992 (0.94–1.01)	0.039
Length of stay	Total (days)		38.21 ± 61.15 (1–402)	48.07 ± 69.9 (3–373)	–	0.200	1.01 (0.94–1.01)	
	Before CDI test		17.1 ± 32.6	18.9 ± 26.4	–	0.636	1.01 (0.99–1.01)	0.636
	After CDI test		21.4 ± 38.3	30.1 ± 59.9	–	0.107	1.01 (0.99–1.01)	0.121

OR: odds ratio; 95% CI: 95% confidence interval; CDI: *Clostridioides difficile* infection; *p*-values statistically significant (*p* < 0.05).

**Table 3:** Association between risk of *Clostridioides difficile* infection and presence of various comorbidities.

Characteristics	Chi-Square Analysis				Conditional Logistic Regression	
	Controls n (%) 273 (75.0)	Cases n (%) 91 (25.0)	Total n (%)	*p*-Value	OR (95% CI)	*p*-Value
Diabetes mellitus	144 (52.7)	49 (53.8)	193 (53.0)	0.856	1.06 (0.62–1.79)	0.839
Hypertension	155 (56.8)	59 (64.8)	214 (58.8)	0.176	1.06 (0.88–2.78)	0.125
Dyslipidemia	61 (22.4)	19 (20.9)	80 (22.0)	0.758	0.9 (0.49–1.66)	0.739
Ulcerative colitis/Crohn disease	10 (3.7)	1 (1.1)	11 (3.0)	0.304	0.27 (0.03–2.22)	0.223
Chronic kidney disease	67 (24.5)	36 (39.6)	103 (28.3)	0.006	2.09 (1.24–3.51)	0.006
Liver disease	29 (10.6)	16 (17.6)	45 (12.4)	0.081	1.79 (0.922–3.5)	0.085
Solid organ transplantation	11 (4.0)	8 (8.8)	19 (5.2)	0.077	2.26 (0.89–15.77)	0.088
Gastrointestinal disease	15 (5.5)	7 (7.7)	22 (6.0)	0.446	1.42 (0.67–3.53)	0.455
Solid cancer or malignancy	40 (14.7)	18 (19.8)	58 (15.9)	0.247	1.42 (0.78–2.6)	0.255
Lymphoma or leukemia	9 (3.3)	7 (7.7)	16 (4.4)	0.076	2.45 (0.88–6.81)	0.086
Congestive heart disease	83 (30.4)	37 (40.7)	120 (33.0)	0.071	1.66 (0.98–2.82)	0.059
Chronic obstructive pulmonary disease	7 (2.6)	5 (5.5)	12 (3.3)	0.175	2.53 (0.7–9.11)	0.156
Nasogastric tube feeding	17 (6.2)	6 (6.6)	23 (6.3)	0.901	1.06(0.4–2.87)	0.898

OR: odds ratio; 95% CI: 95% confidence interval; CDI: *Clostridioides difficile* infection; *p*-values statistically significant (*p* < 0.05).

**Table 4:** Association between *Clostridioides difficile* infection and type of surgery.

Characteristics	Chi-Square Analysis				Conditional Logistic Regression	
	Controls n (%) 273 (75.0)	Cases n (%) 91 (25.0)	Total n (%)	*p*-Value	OR (95% CI)	*p*-Value
Gastrointestinal	33 (12.1)	9 (9.9)	42 (11.5)	0.570	0.79 (0.36–1.75)	0.564
Cardiovascular	19 (7.0)	8 (8.8)	27 (7.4)	0.564	0.127 (0.55–2.99)	0.570
Urology	2 (0.7)	1 (1.1)	3 (0.8)	1.000	1.73 (0.09–30.8)	0.708
General	4 (1.5)	2 (2.2)	6 (1.6)	0.642	1.5 (0.27–8.19)	0.640
Orthopedic	9 (3.3)	3 (3.3)	12 (3.3)	1.000	1 (0.261–3.84)	1
Total no.	67	23	90	–	–	–

OR: odds ratio; 95% CI: 95% confidence interval; CDI: *Clostridioides difficile* infection.

**Table 5:** Association between *Clostridioides difficile* infection and medications.

Characteristics	Chi-Square Analysis				Conditional Logistic Regression	
	Controls n (%) 273 (75.0)	Cases n (%) 91 (25.0)	Total n (%)	*p*-Value	OR (95% CI)	*p*-Value
Proton pump inhibitors	249 (91.2)	85 (93.4)	334 (91.8)	0.509	1.27 (0.53–3.09)	0.593
Ranitidine	68 (24.9)	18 (19.8)	86 (23.6)	0.319	0.74 (0.41–1.34)	0.321
Statin	125 (45.8)	43 (47.3)	168 (46.2)	0.808	1.07 (0.64–1.8)	0.792
Immune suppressant	70 (25.7)	27 (29.7)	97 (26.7)	0.463	1.22 (0.72–2.08)	0.449

OR: odds ratio; 95% CI: 95% confidence interval; CDI: *Clostridioides difficile* infection.

**Table 6:** Multiple regression analysis of factors associated with *Clostridioides difficile* infection among hospitalized patients.

Predictor Variables	Model Parameters		*p*-Value
	Beta	OR (95% CI)	
Age ≥ 70 years	0.6446045	1.90 (1.04–3.45)	0.034
Body mass index	−0.027	0.97 (0.94–1.01)	
Creatinine clearance	0.0047384	1.01 (1.00–1.04)	0.323
Total length of stay total	−0.0018063	1.00 (0.99–1.01)	0.672
Length of stay after CDI test	0.0073645	1.01 (0.99–1.02)	0.214
Hypertension	0.271428	1.31 (0.70–2.50)	0.402
Chronic kidney disease	0.7432566	2.10 (1.02–4.31)	0.043
Liver disease	0.4167027	1.51 (0.72–3.21)	0.276
Solid organ transplantation	1.251179	3.49 (1.20–10.12)	0.021
Lymphoma or leukemia	1.316815	3.73 (1.24–11.22)	0.019
Congestive heart disease	0.1407125	1.15 (0.62–2.12)	0.653
Chronic obstructive pulmonary disease	0.4731865	1.605 (0.472–5.45)	0.449

OR: odds ratio; 95% CI: 95% confidence interval; CDI: *Clostridioides difficile* infection.

**Table 7:** Creation of Clostridium difficile infection risk-prediction model.

Predictor Variables	Model Parameters			
	Beta	OR (95% CI)	No. of Points	Score
Age ≥ 70 years	0.6446045	1.9 (1.04–3.4)	1.9	2
Chronic kidney disease	0.7432566	2.1 (1.02–4.3)	2.3	2
Solid organ transplantation	1.251179	3.5 (1.20–10.1)	3.8	4
Lymphoma or leukemia	1.316815	3.7 (1.24–11.2)	4.1	4

**Table 8:** Model performance at various cutoff points of the developed risk-prediction scores.

Score	TP	FP	TN	FN	SE (%)	SP (%)	PPV	NPV	ACC
≥2	71	149	124	20	78.02	45.4	32.3	86.1	78.02
≥4	40	55	518	51	44	79.9	42.1	81.04	44
≥6	2	264	9	89	2.2	96.7	18.2	0.7	2.2
≥8	1	1	272	90	1.1	99.6	50	75.1	1.1
≥10	0	0	273	91	1.1	99.6			
≥12	0	0	273	91	1.1	99.6			

ACC, accuracy; FN, false negative; FP, false positive; NPV, negative predictive value; PPV, positive predictive value; SE, sensitivity; SP, specificity; TN, true negative; TP, true positive.

## Data Availability

The datasets presented in this article are not readily available because It is owned by the King Abdullah International Medical Research Center and the Ministry of National Guard Health Affairs, in Riyadh, Saudi Arabia. The King Abdullah International Medical Research Center and the Ministry of National Guard Health Affairs should receive requests for access to the datasets.
